# Impact of catastrophizing on pain during orthodontic treatment

**DOI:** 10.1590/2177-6709.25.1.064-069.oar

**Published:** 2020

**Authors:** Eduardo Oliveira da Costa, Marco Nassar Blagitz, David Normando

**Affiliations:** 1 Associação Brasileira de Odontologia - Seção Pará, Curso de Especialização em Ortodontia (Belém/PA, Brazil).; 2 Universidade Federal do Pará, Faculdade de Odontologia, Programa de Pós-Graduação em Odontologia (Belém/PA, Brazil).; 3 Universidade Federal do Pará, Faculdade de Odontologia (Belém/PA, Brazil).

**Keywords:** Pain, Orthodontic, Catastrophizing

## Abstract

**Objective:**

This study proposed to investigate the influence of catastrophizing and others factors related to pain during orthodontic treatment.

**Methods:**

27 patients with 0.022 x 0.028-in Straight-wire brackets were evaluated during alignment and leveling phase with nickel-titanium wires. Visual Analog Scales measured the intensity of orthodontic pain at six moments after a clinical appointment: 6 first hours; 1, 2, 3, 5, and 7 days. Multiple linear regression and stepwise approach assessed the influence of the following variables on pain: catastrophizing, sex, age, duration of treatment, clinical appointment time (morning or afternoon), and wire diameter.

**Results:**

The highest pain intensity was reported 24 hours after activation. These data were used to analyze factors associated with pain level. Age (r = 0.062, *p*= 0.7586), sex (*p*= 0.28), catastrophizing (r = -0.268, *p*= 0.1765), and orthodontic wire diameter (r = 0.0245, *p*= 0.2181) were not correlated with orthodontic pain in the univariate statistics. Catastrophizing was included in the multiple regression model because it was of great interest. Duration of orthodontic treatment (r = 0.6045, *p*= 0.0008) and the time when orthodontic appliance was activated (*p*= 0.0106) showed statistical significant associations with pain, and were also included in the multivariate regression, which showed that about 32% of orthodontic pain could be explained by the duration of treatment (R^2^= 0.32, *p*= 0.0475). Catastrophizing (R^2^= 0.0006, *p*= 0.8881) and clinical appointment time were not significantly associated with pain (R^2^= 0.037, *p*= 0.2710).

**Conclusions:**

Pain after activation of fixed orthodontic appliance is not associated with catastrophizing as well as age, sex, orthodontic wire diameter, and period of activation.

## RESUMO

**Objetivo:**

O presente estudo propôs-se a investigar a influência da catastrofização e outros fatores relacionados à dor durante o tratamento ortodôntico.

**Métodos:**

Foram selecionados 27 pacientes em tratamento com braquetes Straight-wire, *slot* 0,022” x 0,028”, na fase de alinhamento e nivelamento com fios de níquel-titânio. Usando Escalas Visuais Analógicas, mensurou-se a intensidade da dor em seis momentos após a ativação do aparelho: primeiras 6 horas; após 1, 2, 3, 5 e 7 dias. Por meio de regressão linear múltipla e regressão *stepwise*, avaliou-se a influência das seguintes variáveis sobre a dor: catastrofização, sexo, idade, tempo de tratamento, turno de atendimento (manhã ou tarde) e diâmetro do fio ortodôntico.

**Resultados:**

A maior intensidade de dor foi relatada 24 horas após a consulta de ativação do aparelho. Assim, esses dados foram usados para analisar os fatores associados ao nível de dor. Na análise estatística univariada, idade (r = 0,062, *p*= 0,7586), sexo (*p*= 0,28), catastrofização (r = -0,268, *p*= 0,1765) e diâmetro do fio ortodôntico (r = 0,0245, *p*= 0,2181) não estavam associados à dor. Apesar disso, a catastrofização foi incluída no modelo de regressão múltipla, por ser uma variável de maior interesse nesse estudo. A duração do tratamento ortodôntico (r = 0,6045, *p*= 0,0008) e o turno no qual o aparelho foi ativado (*p*= 0,0106) mostraram associação estatisticamente significativa com a dor e também foram incluídos na regressão multivariada, que mostrou que cerca de 32% da ocorrência de dor no tratamento ortodôntico poderiam ser explicados pelo tempo de tratamento (R^2^= 0,32, *p*= 0,0475). Catastrofização (R^2^= 0,0006, *p*= 0,8881) e turno de ativação do aparelho (R^2^= 0,037, *p*= 0,2710) não tiveram influência significativa sobre a ocorrência de dor.

**Conclusão:**

A dor após a ativação do aparelho não está associada à catastrofização, bem como à idade, sexo, diâmetro do fio ortodôntico e turno de atendimento.

## INTRODUCTION

Pain is an unpleasant sensory and emotional experience associated with real or potential tissue damage. The subjectivity of pain brings a great individual variation and dependence of different factors, such as age, sex, emotional state, culture, and previous experiences.^1^

Measuring the subjective perception of pain is a hard task.^2^ The perception of dental pain remains poorly understood.^3^ In Orthodontics, some procedures cause pain and are a major cause of concern for patients and dentists, as well as a reason for treatment discontinuation.^4^ Approximately 90% to 95% of orthodontic patients report pain experiences^5^ and, consequently about 8% of them give up of the treatment. By the way, it is essential to understand the two most important clinical implications of pain: intensity and duration.^4^

The pain resulting from orthodontic movement usually lasts from 2 to 3 days, and its intensity tends to decrease gradually by the fifth or sixth day^6^. In the first 48 hours the pain can be very worrying. About 20% of patients reported waking up at night and most of them describe difficulty in eating. Sometimes they take painkillers and/or anti-inflammatories,^5^ but despite frequent pain experiences, most of patients do not take medications effectively.^7^

Although the clinical importance, the implications related to pain have been poorly investigated in the literature.^8^ Pain seems to be multifactorial and a few number of studies has investigated pain responses after force application and its association to somatic pain response.^9^ A significant influence of psychological factors such as catastrophizing and anxiety has been reported.^10^ Furthermore, there are assumptions that individuals who are likely to react with somatic pain will also react more strongly to dental pain.^7^

Thus, the aim of the present study was to investigate factors associated with pain during orthodontic treatment such as catastrophizing of somatic pain. Age, sex, duration of treatment, the time when the orthodontic appliance was activated (morning or afternoon), and the archwire diameter were also investigated.

## MATERIAL AND METHODS

This study was approved by the Human Research Ethics Committee of the Federal University of Pará under number 088663/2016, and all eligible patients received verbal and written information about the characteristics and objectives of the research.

Twenty-seven voluntary orthodontic patients (19 females and 8 males) were included in this study. The mean age was 27.03 years, with a range of 12-53 years. The following selection criteria were used for subject participation: All participants should be undergoing orthodontic treatment with Straight-wire orthodontic brackets, 0.022 x 0.028-in slot, during alignment and leveling phase with nickel-titanium archwires. The use of extraoral or quadhelix appliances, palatal bar, craniofacial syndromes, ortho-surgical cases, and the inability to understand or complete the questionnaires were adopted as exclusion criteria. 

Immediately after the activation of the orthodontic appliance, the patients were invited to complete the Pain Catastrophizing Scale, as described by Sullivan et al.^11^ and validated in Portuguese by Sehn et al.^12^ Subjects were supervised by one of the investigators in case of doubts, and instructed to recall past experiences of somatic pain, such as headaches, earaches, and stomachaches. The orthodontic pain was not considered at this moment. The following scores were adopted: 0 = minimum, 1 = soft, 2 = moderate, 3 = intense, and 4 = very intense. The final score was given by the sum of the values assigned for each item, ranging from 0 to 52. This instrument is divided in three subscales: Amplification - the thought that something serious may happen; Helplessness - to feel overwhelmed by pain, and Rumination - the act of thinking how much something hurts.

Subjects were invited to complete the questionnaire evaluating pain during orthodontic treatment, by means of Visual Analogue Scales (VAS) in six moments after the activation of the appliance: 6 first hours; 1, 2, 3, 5, and 7 days. The 100-mm scales had respectively the terms “absence of pain” and “maximum pain” on the left and right extremities. The score was measured in millimeters from the left margin to the nearest mark done by the subject. To facilitate interpretation, the numbers were represented by illustrations that showed the sensation of pain that the subjects were going through.

The questionnaire also contained socio-demographic data (age, sex) and clinical annotations (diameter of the orthodontic wire and hour of patient care). Patients were instructed not to take analgesics during the observation period. Otherwise, they should complete the pain questionnaire before using the medication.

### Statistical analysis

The Shapiro-Wilk test analyzed the normality of the pain level variable. The pairwise correlation between pain during orthodontic treatment and quantitative variables was analyzed using the Pearson’s correlation test. The Student’s t-test for two independent samples was applied to evaluate differences in pain level for the variables sex and time of orthodontic appliance activation (morning or afternoon). The variables that showed association with pain level during orthodontic treatment were included in a multiple linear regression and stepwise regression model, to analyze the influence of the independent variables on pain after orthodontic appliance activation (dependent variable) using Bioestat 5.3 software (Mamiraua Institute, Belém, Brazil), with a level of significance of 5%. Statistical analysis also included the descriptive analysis of the questionnaires. 

## RESULTS

The dependent variable (pain level) did not present normal distribution, according to Shapiro-Wilk test (*p*= 0.0098). Thus a logarithmic transformation of the data was applied. Means, standard deviations, or frequencies of all independent variables are shown on Table 1. The mean age of the sample was 27.03 years and the majority of patients were attended in the afternoon. The time of treatment when the questionnaire was applied was 6.5 months on average. Regarding catastrophizing, it was verified that the total scale score varied between 8 and 37, and the mean was 22.48.

**Table 1:** Descriptive statistics, t-test (dichotomous variables), and correlation between pain level (VAS) and the independent variables.

Variables						
	Mean/ f	SD	Min	Max	r	P
Orthodontic pain	3.4	3.06	0	9	-	-
Catastrophizing	22.48	8.30	8	37	-0.268	0.1765
Duration of treatment (months)	6.51	6.028	0	20	0.6045	0.0008
Archwire diameter (Up + Lw)	0.023	0.011	0.01	0.05	0.245	0.2181
Age of the patients	27.03	12.54	12	53	0.062	0.7586
Sex* (Male/Female)	8/19	-	-	-	-	0.2833
Hour on appliance activation* (Morning/ Afternoon)	10/17	-	-	-	-	0.0106

Level of significance: p ≤ 0.05; *f: Dichotomous variables.

**Figure 1:**
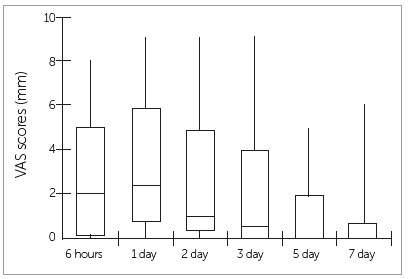
Intensity of pain after orthodontic appliance activation.

The highest intensity of pain was reported on the first day after orthodontic appliance activation (Fig. 1). Pain levels tended to decrease after that, although some patients still reported pain for a longer period. Thus, it was decided to use the pain intensity values on the first day to perform the analysis of the factors associated with the level of pain.

Three patients have reported the use of analgesics during the observation period, and two of these reports were 24 hours after the device’s activation. 

In the univariate analysis, the variables age (r = 0.062, *p*= 0.7586), sex (*p*= 0.28), catastrophizing (r = -0.268, *p*= 0.1765), and archwire diameter (r = 0.0245, *p*= 0.2181) showed no significant association with pain during orthodontic treatment. However, catastrophizing was included in the multivariate model because it was the variable of interest (Table 1). The time that patient was under orthodontic treatment (r = 0.6045, *p*= 0.0008) and the hour of the day in which the appliance was activated (*p*= 0.0106) were also included in the multiple regression model.

Multiple linear regression showed that about 32% of the occurrence of orthodontic pain could be explained by the duration of orthodontic treatment (R^2^= 0.32, *p*= 0.0475, Table 2). A positive relationship indicates that a longer time with orthodontic appliance leads to a greater report of pain. Catastrophizing (R^2^= 0.0006, *p*= 0.8881) and hour of appliance activation(R^2^= 0.037, *p*= 0.2710) variables showed no significant influence on pain level (*p*> 0.05).

**Table 2:** Multiple Linear Regression and Stepwise method of variables associated with orthodontic pain (F=4.28; p=0.0152; R^2^=0.3584).

Variables	Regression Coefficient	Multiple Regression	
		R^2^	P
Catastrophizing	-0.001	0.0006	0.8881
Duration of treatment	0.023	0.3208	0.0475
Hour of appliance activation	-0.156	0.0370	0.2710

## DISCUSSION

Pain caused by orthodontic forces is reported to be associated with discontinuation of orthodontic treatment. A higher pain intensity on the first day after the activation of the device was observed in this study and in previously reported findings.^5^ ^,^ ^7^ However, studies have reported that pain may persist even after removal of the appliance.^13^

It is recognized the catastrophizing role in potentiating pain, increasing its perception and decreasing its tolerance. Catastrophizing has a considerable influence on pain experiences during dental treatment.^14^ In orthodontics, an association between catastrophizing and pain caused by orthodontic separators has been reported.^10^ ^,^ ^15^ Patients using separators who would still initiate orthodontic movement were not experiencing pain as often as those patients whose orthodontic appliance is activated monthly. Furthermore, the intensity of pain caused by separators may be considerably greater than the pain caused by NiTi archwires during alignment. The present results showed that catastrophizing is not associated with pain level caused by orthodontic movement. Previous studies reported that the relationship between catastrophizing and pain may be moderated by the stage of chronicity.^16^ Studies have shown that the subscales of the Pain Catastrophizing Scale may be important predictors of the severity of disability in patients who have been suffering from chronic pain for years.^17^ ^,^ ^18^

The present findings also indicated that the duration of orthodontic treatment seems to have a negative impact on pain level. Based on these findings, it is suggested that subjects under orthodontic treatment for a longer time may be more intolerant and have higher expectations of completing the treatment. Considering the different factors that modulate pain experiences and the great individual variation in their perception, it is important to recognize patients’ emotional state as well as their motivation to continue and finish orthodontic treatment.^4^ ^,^ ^8^

The present study could not find a significant association between pain level and sex or age. The literature is controversial regarding these topics. With regard to sex, some studies indicate that women are potentially more likely to report pain,^2^ ^,^ ^4^ ^,^ ^7^ while others reported no difference.^19^ For the variable age, although the absence of a relationship between age and pain has been previously reported,^7^ ^,^ ^19^ it has been described that adults perceive more pain than young patients during orthodontic treatment.^2^ ^,^ ^20^

A study that compared the use of 2 x 4 appliances to a full appliance in one or in both arches did not show statistical differences on the frequency and intensity of pain reported by orthodontic patients.^5^ Also, no difference was observed for pain after insertion of initial archwire of two different sizes (NiTi 0.014-in and 0.016-in).^21^ These findings are supported by the results found in the present study. 

Regarding the hour of the day in which the orthodontic appliance was activated (morning or afternoon), no influence on the intensity of pain was observed. However, there are reports showing that pain tends to be more intense in the late afternoon and at night.^22^

There are interesting clinical implications for the results observed in this study. The findings suggest that interventions that incorporate the subjectivity of the patient may have beneficial effects in the clinical setting. The possibility of quantitatively measuring pain brings relevant information about the determination of need, efficiency, and treatment time. It also guides the dentist’s therapeutic behavior, minimizing pain and/or discomfort of the patient during treatment.^23^ This understanding can help to improve resource management, such as patient education, motivation, and effective use of pharmacological agents for pain relief. However pain is multifactorial and showed a high level of variability with a few number of aspects that can explain this variability.

The limitations found in this study should also be considered. The subjective nature of pain and the wide range of differences between individuals make it difficult to measure even when used similar criteria. Another limiting factor is the great variability of archwires used in different patients, whose standardization becomes difficult to be modeled. It is still worth to take into account the transversal design of these findings regarding archwire sequence. Future research should reinforce the data analyzed in the present study, such as longitudinal studies that evaluate pain reports, due to the type of alloy and the archwire size, as well as the real effect of the treatment time on pain produced by the orthodontic appliance. These studies are essential to better understand patient’s response to pain in orthodontics. Despite these limitations, the results bring interesting information about an non-investigated question that is the relationship between orthodontic pain and somatic pain. 

## CONCLUSIONS

» The pain experience during orthodontic treatment has a high variability and is more intense one day after the activation of the orthodontic appliance.
» No significant association was found between pain during orthodontic treatment and somatic pain, as well as to the variables age, sex, orthodontic archwire diameter, and hour of patient care (morning or afternoon).
